# Expression of Drug-Resistant Factor Genes in Hepatocellular Carcinoma Patients Undergoing Chemotherapy with Platinum Complex by Arterial Infusion 

**DOI:** 10.3390/pharmaceutics2030300

**Published:** 2010-09-09

**Authors:** Tomoya Sakurada, Masaharu Yoshikawa, Masahiko Sunaga, Eriko Kobayashi, Nobunori Satoh, Osamu Yokosuka, Shiro Ueda

**Affiliations:** 1 Department of Drug Information Communication, Graduate School of Pharmaceutical Sciences, Chiba University, 1-8-1 Inohana, Chuo-ku, Chiba, 260-8675, Japan; 2 Department of Medicine and Clinical Oncology, Graduate School of Medicine, Chiba University, 1-8-1 Inohana, Chuo-ku, Chiba, 260-8675, Japan; 3 Department of Gastroenterology, Chiba Central Medical Center, 1835-1 Kasoricho, Wakaba-ku, Chiba, 264-0017, Japan; 4 Department of Clinical Education and Research, Graduate School of Pharmaceutical Sciences, Chiba University, 1-8-1 Inohana, Chuo-ku, Chiba, 260-8675, Japan

**Keywords:** canalicular multispecific organ anion transporter, chemotherapy, drug-resistance, hepatocellular carcinoma, metallothionein, platinum complex

## Abstract

This study investigated gene expression of drug resistance factors in biopsy tissue samples from hepatocellular carcinoma (HCC) patients undergoing chemotherapy by platinum complex. Liver biopsy was performed to collect tissue from the tumor site (T) and the non-tumor site (NT) prior to the start of treatment. For drug-resistant factors, drug excretion transporters cMOAT and MDR-1, intracellular metal binding protein MT2, DNA repair enzyme ERCC-l and inter-nucleic cell transport protein MVP, were investigated. The comparison of the expression between T and NT indicated a significant decrease of MT2 and MDR-1 in T while a significant increase in ERCC-1 was noted in T. Further, expression was compared between the response cases and non-response cases using the ratios of expression in T to those in NT. The response rate was significantly low in the high expression group when the cutoff value of cMOAT and MT2 was set at 1.5 and 1.0, respectively. Furthermore, when the patients were classified into A group (cMOAT ≧ 1.5 or MT2 ≧ 1.0) and B group (cMOAT < 1.5 and MT2 < 1.0), the response rate of A group was significantly lower than B group when we combined the cutoff values of cMOAT and MT2. It is considered possible to estimate the therapeutic effect of platinum complex at a high probability by combining the expression condition of these two genes.

## 1. Introduction

Hepatocellular carcinoma (HCC) is a cancer which is derived from liver cells and originates in the liver. It is known that HCC occurs with chronic hepatitis and cirrhosis attributable to hepatitis B or C virus as the base. In the case of local presence of HCC, surgical resection and localized treatment are applicable but chemotherapy with anticancer agents is indicated for multiple progressive cases. Concerning the treatment of HCC using anticancer agents, hepatic arterial infusion chemotherapy (HAI) is the method generally conducted at present. As for the drugs used in the treatment of HCC, anthracyclin was primarily used in the past, but platinum complex and pyrimidine fluoride have come to be used recently. The present study focused on a platinum complex frequently used in the treatment of HCC and the relevant drug-resistant factors were investigated. 

Many reports on the drug-resistant factors related to platinum complex have cited the decrease in intracellular accumulation, enhanced inactivation of drug and enhanced DNA repair as the main resistance mechanisms [1,2]. The excessive expression of drug excretion transporters is conceivable as a cause of the decreased intracellular accumulation. Concerning the multiple drug-resistant factors, it is reported that p-glycoprotein, an ABC transporter coded by multi-drug resistance-1 (MDR-1), is a multi-drug-resistance factor involved in the resistance of a wide range of anticancer agents [3]. As for the drug excretion transporter involved in the resistance to platinum complex, most reports suggest canalicular multispecific organ anion transporter (cMOAT) which is a type of ATP binding cassette (ABC) transporter [4,5,6,7]. cMOAT is an ABC transporter that transports the matrix using ATP as the driving force and is considered to move the intracellular platinum complex to outside the cell. It is reported that metallothionein (MT) [8,9,10], gluthathione (GSH) [11,12] and glutathione-S-transferase (GSTπ) [13] are mainly involved in the enhanced inactivation of drug. Both MT and GSH possess a thiol group. The inactivation of platinum complex occurs by the substitution of this thiol group with an active group of platinum complex. GSTπ is an enzyme which catalyzes the inactivation of drug by GSH. The increase in the intracellular concentrations of these proteins is considered to produce the resistance to platinum complex [9,10,12]. However, it is reported that in HCC the expression of GST is low based on immunohistological examination [14]. Also it is reported that nucleotide excision repair (NER) is closely involved in the enhanced DNA repair. It is considered that after migration into the cell, the platinum complex binds to intranuclear DNA to form an adduct and this inhibits the transcription and replication of DNA to demonstrate cytotoxicity. NER is considered as the most important pathway in the removal of this DNA adduct [15]. ERCC-1, a DNA repair enzyme related to NER, is reported to be a resistant factor for platinum complex [16,17,18,19]. In addition to the above-mentioned pathways, major vault protein (MVP), a platinum complex-resistant factor, has been recently investigated [20,21,22]. MVP is a protein which forms vault protein that is reported to be involved in the inter-nucleoplasmic transport and is considered to transport the intranuclear drug to outside of the nucleus [23]. As for other drug resistance mechanisms, the involvement of mismatch repair defect [24,25], enhanced drug excretion transporter ATP7B expression [26,27], and cancer-inhibiting gene p53 defect [25] have been reported. 

However, the relationship between drug-resistant factor expression in clinical samples and the effect of treatment with platinum complex has not been investigated with regard to HCC. Accordingly, this study aimed at predicting the therapeutic effect of platinum complex according to the expression of cMOAT, MT2, ERCC-1, and MVP, which have been frequently reported as resistance factors to platinum complex as well as that of the multiple drug-resistant factor MDR1. The characteristics of resistant factor gene expression were investigated in relation to the therapeutic effect in clinical cases in which HAI was performed using platinum complex, and the possibility of predicting the therapeutic effect on the basis of expression of drug resistant factors was investigated. 

## 2. Experimental Section

### 2.1. Hepatocellular carcinoma sample

The subjects of this study were 35 HCC patients who received the HAI with platinum complex alone at Chiba University Hospital from 1999 to 2000. This study was approved by the Ethics Committee of Chiba University Hospital. After informed consent was obtained and prior to the start of treatment, liver biopsy was performed under ultrasonic imaging method using a 21G thin needle to collect tissue from the tumor site (T) and a non-tumor site (NT). Immediately after collection, the tissue was frozen with liquid nitrogen and stored at -120 °C. Table 1 summarizes the therapeutic responses of the treatments for the background of HCC patients. In each parameter, there was no factor indicated to be related to therapeutic effect. The treatment was responsive in 17 and nonresponsive in 18 cases for a response rate of 48.6% (17/35).

Also, the pretreatment included a milli-platinum administration in one case, epirubicin + cisplatin administration in five cases, epirubicin administration in 11 cases and chemo-embolisation in 10 cases. 

**Table 1:** Patient characteristics and clinical data.

Parameter	Total number	Responders	Nonresponders	p value
Age (years old)	67.6 ± 6.8*			
Gender				
Males	30	13	17	0.151
Females	5	4	1	
Virus				
HBV(+)	1	0	1	0.367
HCV(+)	33	17	16	
None	1	0	1	
Stage				
Ⅲ	32	14	18	0.103
ⅣA	3	3	0	
Child's classification				
A	17	11	6	0.158
B	16	5	11	
C	2	1	1	
Noncancerous tissue				
Cirrhosis	34	16	18	0.486
Chronic hepatitis	1	1	0	
Chemotherapy				
CBDCA	24	10	14	0.200
CDDP	11	7	4	

Data indicate number of patients. *Data is mean ± SD.

### 2.2. cDNA standards

For external standards, the drug resistance factors and housekeeping gene (GAPDH) were cDNA transcripts of RNA transcribed from Human Liver Total RNA (Takara Bio, Shiga, Japan). Serial dilutions of RNA were made in diethylpyrocarbonate treated water and a known amount of RNA transcripts were used for cDNA synthesis. The random pd(N)6 primer (100 ng) (Amersham Pharmacia Biotech Inc., Piscataway, NJ, USA) and the sample were denatured at 65 °C for 1 min and cooled on ice. After RNA extraction, cDNA was prepared by reverse transcription using Omniscript Reverse Transcription (RT) kit (QIAGEN K.K., Tokyo) and Rnase inhibitor (Roche Diagnostics GmbH, Mannheim, Germany). The reaction was performed on a standard polymerase chain reaction (PCR) apparatus at 37 °C for 60 min and the samples were denatured thereafter at 93 °C for 5 min. The cDNAs were stored at -70 °C.

### 2.3. RNA extraction from the tissue samples

RNA was extracted from tissue samples using the RNase free DNase kit (QIAGEN K.K.) and total RNA extraction was performed using RNeasy mini kit (QIAGEN K.K.) according to the manufacturer’s instructions. DNase was used to digest and remove genomic DNA contamination. The amount of RNA was quantified with a GeneQuant pro personal spectrophotometer (Biochrom Ltd., Cambridge, UK) at a wavelength of 260 nm (A260). Purity of total RNA was determined by the A260/A280 ratio. The integrity of RNA samples was confirmed by electrophoresis on a 2% agarose gel. In order for the RNA sample to be considered for experiments, an A260/A280 ratio of between 1.8 and 2.1 was necessary. After RNA extraction, cDNA was prepared by reverse transcription using the Omniscript RT kit. 

### 2.4. Primers and probes

The synthesis of primer and probe was performed by Nihon Gene Research Laboratories Inc. (Sendai). Table 2 shows the base sequence of the primer and probe. Probes designated as LC contained an acceptor dye LightCycler Red 640 covalently attached to the 5’ end. The FL probes contained the donor dye Fluorescein (FL) at the 3’ end.

### 2.5. Real-time quantitative PCR

Real-time quantitative RT-PCR (qRT-PCR) was performed with the LightCycler Faststart DNA Master hybridization probes (Roche Diagnostics GmbH, Mannheim, Germany), according to the manufacturer's instructions. Each reaction was carried out in a total volume of 20 μL in glass capillaries, containing 5 μL of cDNA sample, 25 mM MgCl_2_, 10% LightCycler Faststart DNA Master hybridization probes buffer (Taq DNA polymerase, reaction buffer, deoxynucleotide triphosphate mix and LightCycler Faststart enzyme), 10 pmol each primer, and for the probe reactions, 4 pmol (Flu) and 8 pmol (LC). The cDNA sample was denatured at 95 °C for 10 min and then added to the capillaries. The reaction was carried out using the following conditions for GAPDH, cMOAT and MDR-1: 40 cycles of 95 °C for 10 s/60 °C for 10 s/72 °C for 9 s with a single fluorescence detection point at the end of the relevant annealing or extension segment. For ERCC-1 and MVP the annealing temperature was 62 °C. For ERCC-1 and MT2 the extension time was 8 s and 7 s, respectively. After this, one cycle of melting curve from 70 to 95 °C by a transition rate of 0.2 °C/s with continuous detection of fluorescence, was performed. For ERCC-1 and MVP, one cycle of the melting curve from 70 to 99 °C by a transition rate of 0.1 °C/s was performed. The temperature transition rate for all amplifications was 20 °C/s. Negative controls were concomitantly run to confirm that the samples were not cross-contaminated. A sample with 1 μL diethylpyrocarbonate-treated water in place of RNA was concomitantly examined for each of the reaction units described above. Analysis was carried out with the LightCycler 3.5 software (Roche Diagnostics GmbH, Mannheim, Germany). A positive control consisting of cDNA from Human Liver Total RNA (Takara Bio) was also added to each LightCycler run. In addition, a standard curve was obtained using serial dilutions of positive control. The standard curve, plotted cycle number *versus* log concentration, was considered reliable when -0.09 > r ≧ -1.00. The drug resistance factor expressions were indicated as a relative ratio to GAPDH. Though substantial dispersion was observed depending on the sample, total RNA yield was 11.29 ± 2.18 μg * (0.20–67.7 μg) in T, and 7.26 ± 2.96 μg * (0.20–90.0 μg) in NT (*: mean ± SE). Since all of the subjects of this study were patients with progressive HCC, the total RNA yield of some samples was not sufficient for the determination of all five resistant factors; MDR-l was investigated in 33 cases and MVP in 30 cases.

**Table 2:** The primers and probe sequences for GAPDH and the drug resistance factors.

Gene		Sequence	Position	GenBank Access No.	Size (bp)
GAPDH	Forward primer (5'→3')	TGAACGGGAAGCTCACTGG	732	M33197	307
	Reverse primer (5'→3')	TCCACCACCCTGTTGCTGTA	1019		
	probe-Flu (5'→3')	TCAACAGCGACACCCACTCCT	918		
	Probe-LC (5'→3')	CACCTTTGACGCTGGGGCT	940		
cMOAT	Forward primer (5'→3')	CGACCCTTTCAACAACTACT	4223	E15807	248
	Reverse primer (5'→3')	TCGTCTGAATGAGGTTGTCT	4451		
	probe-Flu (5'→3')	AGGTTGCCACCAGCCTCTGTCACTT	4299		
	Probe-LC (5'→3')	GTGGGATAACCCAAGTTGCAGGCT	4324		
MDR-1	Forward primer (5'→3')	GGCAAAGAAATAAAGCGACT	3419	AF016535	201
	Reverse primer (5'→3')	TTTATTAGGCAGTGACTCGA	3590		
	probe-Flu (5'→3')	GGGTGGTGTCACAGGAAGAGATTGTG	3530		
	Probe-LC (5'→3')	GGGCAGCAAAGGAGGCCAACATA	3557		
MT2	Forward primer (5'→3')	CACCTCCTGCAAGAAAAG	79	X97260	186
	Reverse primer (5'→3')	ACGGTCAGGGTTGTACAT	247		
	probe-Flu (5'→3')	CCCTTTGCAGATGCAGCCCTG	105		
	Probe-LC (5'→3')	GCACACTTGGCACAGCCCACA	137		
ERCC1	Forward primer (5'→3')	CGACGTAATTCCCGACTATG	515	AF001925	194
	Reverse primer (5'→3')	ACATCTTAGCCAGCTCCTTG	689		
	probe-Flu (5'→3')	AGGCGAAGTTCTTCCCCAGGCTC	593		
	Probe-LC (5'→3')	GCAGCCGCCCATGGATGTAGTCT	617		
MVP	Forward primer (5'→3')	GGCTTTGAGACCTCGGAA	1883	NM_017458	236
	Reverse primer (5'→3')	TCCTGCTCCAGTCTCTGA	2102		
	probe-Flu (5'→3')	GGTCCTCTGATCCACAGGCTCCACT	1970		
	Probe-LC (5'→3')	ACTGCACGTCCACACTGCTGACCA	1996		

### 2.6. Treatment and therapeutic effect

Carboplatin (300 mg) was infused with an interval of 4 weeks between treatments in 24 cases and cisplatin (50 mg) was infused in a similar administration interval in six cases. The anticancer agent was infused through a catheter retained at the proper hepatic artery level. Regarding the five remaining cases, cisplatin (100 mg) was infused (one shot arterial infusion: TAI) once or twice at the proper hepatic artery level at the time of angiography. The mean number of treatment times was 7.9. The dose was reduced when any adverse reaction such as bone marrow inhibition occurred. The therapeutic effect was judged according to the response evaluation criteria in solid tumors (RECIST) [28]. CR and PR cases were assessed as responders while SD and PD cases were assessed as non-responders.

### 2.7. Statistic analysis

Wilcoxon signed-rank test was employed for comparison of expression between T and N. The correlation between the resistant factors was assayed by Spearman’s correlation coefficient by rank test. For comparison of responders with non-responders, Mann-Whitney's U test was employed. The cut-off values of the T/NT ratio were set at levels higher than the cut-off values and at the levels at which the probability is actually nonresponsive and the lowest probability actually responsive become the maximum. X2 test and Fischer's exact probability test were used for comparison of patient’s background and therapeutic effect, and for the comparison of the response rate classified by the resistant factor cut-off value. Statistical significance was defined as a P value of less than 0.05.

## 3. Results and Discussion

### 3.1. Expression of each resistant factor gene in T and NT

**Figure 1:**
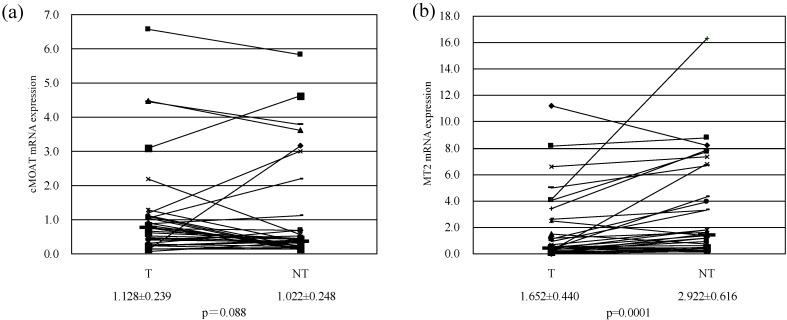
**(a)** cMOAT mRNA expression in T and corresponding NT (N = 35). **(b)** MT2 mRNA expression in T and corresponding NT (N = 35). Values are mean ± SE. Bar: median value.

Expression of each resistant factor gene mRNA in T and NT is shown in Table 3. The expression in T increased in 24 cases and decreased in 11 cases in comparison with NT, and the cMOAT expression in T tended to show dispersion (Figure 1-a). According to other reports that investigated the expression of cMOAT in resected liver tissue of HCC patients, the expression in T tended to show more dispersion in comparison with that in NT [29]. This was also true with the patients treated in our department. These results indicated that the cMOAT expression in T demonstrates the presence of individual difference as well as the versatile changes in comparison with the status in NT. Furthermore, compared with the status in NT, the MDR-l expression in T was significantly decreased (p = 0.009). Regarding the expression of MDR-1 in T in HCC, some have reported an increase [30] while others reported no change [29,31] or even a decrease [32,33,34]. As such, there is no consensus. However, there is also a report which found that the expression of p-glycoprotein in T decreased along with the decrease in histological differentiation [32,35]. Also, regarding the expression of MT2 in resected liver tissue of HCC patients, there are similar reports on histological differentiation [36,37]. The MT2 expression was significantly decreased in T in comparison with that in NT (p = 0.0001) (Figure 1-b). Since all of the subjects of this study were patients with progressive HCC, the decreased histological differentiation in T is considered to have been involved in the decrease in the expression of MDR-1 and MT2. The ERCC-1 expression was significantly increased in T in comparison with that in NT (p = 0.008). It has been reported that the ERCC-1 expression in T in resected liver tissue of HCC patients is increased in comparison with the status in normal liver tissue and NT, and it has also been reported that the expression of ERCC-1 is increased in NT complicated with cirrhosis in comparison with normal liver tissue [38,39]. Accordingly, the expression of ERCC-1 is considered to increase in the progression from normal liver through cirrhosis to HCC, and the increased expression is involved in the anticancer drug resistance in HCC. In comparison with the status in NT, MVP expression in T was increased in 18 cases and decreased in 21 cases but there was no significant difference in the expression between T and NT. Considering that MVP is particularly known to be involved in biological defense and it is induced by various substances that demonstrate cytotoxicity, other than anticancer agents, it is considered that the MVP expression tended to show substantial dispersion between the individuals. It was also reported that the MVP expression shows no correlation between T and NT and no specific tendency (increase, decrease, *etc.*) in HCC patients [40]. This was also true for the patients treated in our department.

**Table 3:** mRNA expression of each resistance factor gene in T and NT.

	T	NT	p value
cMOAT ( N=35 )	1.128 ± 1.419	1.022 ± 1.479	0.088
MDR-1 ( N=33 )	0.712 ± 0.932	1.281 ± 1.701	0.009
MT2 ( N=35 )	1.652 ± 2.604	2.922 ± 3.646	0.0001
ERCC-1 ( N=35 )	0.024 ± 0.028	0.015 ± 0.011	0.008
MVP ( N=30 )	0.012 ± 0.010	0.009 ± 0.005	0.360

Values are mean ± SD.

The correlation of the expression of each resistant factor gene between T and NT is shown in Table 4. In the cMOAT, the tendency for a weak correlation was observed in the expression between T and NT (r = 0.283), but it was not significant (p = 0.099). MDR-1, MT2 and ERCC-1 indicated a strong correlation in the expression between T and NT (r > 0.7, p < 0.0001, respectively), suggesting that the individual tendency in NT is also maintained in T. Concerning the MVP, there was no correlation between the expression of T and NT.

**Table 4:** Correlation of the mRNA expression of each resistant factor gene between T and NT.

	Correlation coefficient (rs)	p value
cMOAT ( N = 35 )	0.283	0.099
MDR-1 ( N = 33 )	0.833	<0.0001
MT2 ( N = 35 )	0.769	<0.0001
ERCC-1 ( N = 35 )	0.808	<0.0001
MVP ( N = 30 )	0.147	0.427

### 3.2. Relation of drug-resistant factor expression to therapeutic effect

The therapeutic effect of the platinum complex is shown in Table 5. The treatment was responsive in 17 and nonresponsive in 18 cases for a response rate of 48.6% (17/35). The comparison of drug-resistant factor gene expression between T and NT indicated a significantly lower T/NT ratio of cMOAT in the responders than in the nonresponders. Therefore, the expression of cMOAT is assumed to be closely involved in the therapeutic effect of the platinum complex in HCC. As for MT, it was reported that the expression of MT protein was significantly lower in the response group than in the nonresponse group when investigated by immunohistological staining in the HCC patients treated with carboplatin [37]. No significant difference was observed in the T/NT ratio of MT2 between the responders and nonresponders. However, there were four cases whose T/NT ratio was as high as 5.0 or more, and all of them were nonresponders. As for MDR-1, one report stated that the cell line prepared through transfection of MDR-l gene did not demonstrate resistance to cisplatin [41]. Based on this report, it is assumed that cisplatin does not serve as the matrix for p-glycoprotein. In this regard, there was no relation between the expression of MDR-l and the therapeutic effect of platinum complex in this study, indicating that MDR-1 is not related to the resistance to platinum complex. As for ERCC-l, there was a study in ovarian cancer patients that found that the expression in the non-responders to the treatment with platinum complex was significantly higher than that in the responders [42,43]. The results of this study indicated a significant increase in the ERCC-l expression in HCC in comparison with NT but no relation was observed between the expression and therapeutic effect. Regarding T/NT ratio of MVP, there was no significant difference between responders and nonresponders.

**Table 5:** Therapeutic effect and T/NT ratio of each resistance factor gene mRNA expression level.

T/NT ratio	responders		nonresponders		No. of responders / nonresponders	p value
	median	range	median	range		
cMOAT (N = 35)	1.067	0.46-3.97	2.595	0.04-9.17	17 / 18	0.024
MDR-1 (N = 33)	0.813	0.02-2.48	0.754	0.02-7.56	15 / 18	1.000
MT2 (N = 35)	0.361	0.02-0.93	0.336	0.03-1.99	17 / 18	0.987
ERCC-1 (N = 35)	1.237	0.48-9.74	1.307	0.66-4.35	17 / 18	0.779
MVP (N = 30)	1.086	0.33-2.98	1.057	0.38-30.62	17 / 13	0.900

Therapeutic effect for cut-off value by the T/NT ratio of cMOAT and MT2 is shown in Table 6. With cut-off values set for cMOAT at 1.5 or MT2 at 1.0, the patients were classified into high (A group; cMOAT ≧ 1.5 or MT2 ≧ 1.0) and low expression groups (B group; cMOAT < 1.5 and MT2 < 1.0), respectively.

The response rate in the high expression group was significantly lower than that in the low expression group (cMOAT; p = 0.003, MT2; p = 0.011). As a means to predict the therapeutic effect, whether or not it would be possible to estimate the response and nonresponse at a higher probability was investigated by combining the cut-off values of cMOAT and MT2. While 14 of the 16 patients in A group were non-responders, 15 of the 19 in B group were responders. As a result, it was possible to estimate the therapeutic effect of platinum complex at a high probability (p < 0.0001).

**Table 6:** Relationship between cMOAT and MT2 mRNA expression and therapeutic effect.

Expression of cMOAT and MT2 mRNA (T/NT ratio)	No. of	No. of	p value
	responders	nonresponders	
cMOAT ≧ 1.5 (n = 13)	2	11	0.003
cMOAT < 1.5 (n = 22)	15	7	
MT2 ≧ 1.0 (n = 6)	0	6	0.011
MT2 < 1.0 (n = 29)	17	12	
Group A (n = 16)	2	14	<0.0001
Group B (n = 19)	15	4	
Total	17	18	
Group A: cMOAT ≧ 1.5 or MT2 ≧ 1.0			
Group B: cMOAT < 1.5 and MT2 < 1.0			

ERCC-l and MVP, whose involvement in the resistance to platinum complex has been reported in other cancers, did not demonstrate any definite relation in HCC. The reasons conceivable are the use of carboplatin in a majority of subjects of this study while the majority of the previously reported patients were treated with cisplatin. Because of the complicated stereoscopic structure in comparison with cisplatin, carboplatin is assumed to demonstrate a different reaction to resistant factors. Further, it was reported that human organic cation transporter (SLC22A1-3) is present among the transporters and that SLC22A1-3 transports cisplatin but not carboplatin [44]. Therefore, considering that the contribution ratio of each resistant factor to the resistance to carboplatin is different from that to cisplatin and that the possible presence of other resistant mechanisms is not totally deniable, it is necessary to investigate the resistance mechanism specific to carboplatin in the future. Also, there was little quantity of specimen collection by aspiration, from which levels of drug resistant genes expression were normalized to GAPDH levels. The potency that aspiration causes hypoxia by hemostasis such as surgery specimen is lower. However, with hypoxia, expression level of house-keeping gene may change. It is theoretically possible that GAPDH levels in samples may vary in samples, which is a potential limitation of this study and may alter findings. Also, since only mRNA was analyzed in this study, not protein, this is also a limitation of the study.

## 4. Results and Discussion

An explanation for the poor therapeutic effect of anticancer agents in HCC may be the possible involvement of other cell membrane transporters and intracellular protein, and mRNA expression of DNA repair enzymes that was not investigated in this study. However, it is considered that enhanced DNA repair by ERCC-1 is involved to some extent in addition to the varied expression patterns of cMOAT, MT2, MDR-1 and MVP in T and NT of individual cases. 

Furthermore, cMOAT and MT2 are closely involved in the therapeutic effect of platinum complex in HCC. In this regard, it is considered possible to estimate the therapeutic effect of platinum complex at a high probability by combining the cMOAT and MT2 gene expression conditions. 

In the future, it will be necessary to investigate the meaning of these drug-resistant factors in the prediction of therapeutic effect by conducting a prospective study.
